# Characterizing Motor Control of Mastication With Soft Actor-Critic

**DOI:** 10.3389/fnhum.2020.00188

**Published:** 2020-05-26

**Authors:** Amir H. Abdi, Benedikt Sagl, Venkata P. Srungarapu, Ian Stavness, Eitan Prisman, Purang Abolmaesumi, Sidney Fels

**Affiliations:** ^1^Electrical and Computer Engineering Department, University of British Columbia, Vancouver, BC, Canada; ^2^Department of Prosthodontics, University Clinic of Dentistry, Medical University of Vienna, Vienna, Austria; ^3^Department of Computer Science, University of Saskatchewan, Saskatoon, SK, Canada; ^4^Department of Surgery, University of British Columbia, Vancouver, BC, Canada

**Keywords:** mastication modeling, reinforcement learning, soft actor-critic, inverse dynamics, jaw, motor control, musculoskeletal modeling, computational biomechanics

## Abstract

The human masticatory system is a complex functional unit characterized by a multitude of skeletal components, muscles, soft tissues, and teeth. Muscle activation dynamics cannot be directly measured on live human subjects due to ethical, safety, and accessibility limitations. Therefore, estimation of muscle activations and their resultant forces is a longstanding and active area of research. Reinforcement learning (RL) is an adaptive learning strategy which is inspired by the behavioral psychology and enables an agent to learn the dynamics of an unknown system via policy-driven explorations. The RL framework is a well-formulated closed-loop system where high capacity neural networks are trained with the feedback mechanism of rewards to learn relatively complex actuation patterns. In this work, we are building on a deep RL algorithm, known as the Soft Actor-Critic, to learn the inverse dynamics of a simulated masticatory system, i.e., learn the activation patterns that drive the jaw to its desired location. The outcome of the proposed training procedure is a parametric neural model which acts as the brain of the biomechanical system. We demonstrate the model's ability to navigate the feasible three-dimensional (3D) envelope of motion with sub-millimeter accuracies. We also introduce a performance analysis platform consisting of a set of quantitative metrics to assess the functionalities of a given simulated masticatory system. This platform assesses the range of motion, metabolic efficiency, the agility of motion, the symmetry of activations, and the accuracy of reaching the desired target positions. We demonstrate how the model learns more metabolically efficient policies by integrating a force regularization term in the RL reward. We also demonstrate the inverse correlation between the metabolic efficiency of the models and their agility and range of motion. The presented masticatory model and the proposed RL training mechanism are valuable tools for the analysis of mastication and other biomechanical systems. We see this framework's potential in facilitating the functional analyses aspects of surgical treatment planning and predicting the rehabilitation performance in post-operative subjects.

## 1. Introduction

The stomatognathic or masticatory system is one of the most complex functional units in the human body. It is characterized by a multitude of skeletal components, teeth, soft tissues, muscles, tendons, ligaments, and fibrous disks. The mandible is at the heart of this complex and is connected to the skull via the mandibular condyles. The condyles of the mandible are located inside the glenoid fossa of the temporal bone and the collective of them forms the temporomandibular joint (TMJ), hence the name. The TMJ is a ginglymoarthrodial joint and enables the mandible to exhibit rotational and translational movements constrained by the passive tensions of the ligaments, muscles, and other passive factors (Gallo et al., 2000). Two TMJs form a functional masticatory system which enables the mandible to rotate and translate with six degrees of freedom across its limited domain of motion (Drake et al., 2014). The TMJs are among the most utilized joints in the human body and play an essential role in chewing and speaking functions.

During mastication, like any other biomechanical routine, a set of time-varying neural and muscular activations work in unison to enable kinematics. Motor control is a highly complex process that involves the nervous and musculoskeletal systems. The peripheral neurons innervate the muscles. Upon excitation of the neural pathways, the skeletal muscles are activated which generate forces to actuate the joints. Neural excitation patterns and, in turn, the muscle activation trajectories are often unknown.

An electromyograph is a highly sensitive voltmeter that detects the electric potential from the transmembrane current of the muscle fibers and a common research tool in many disciplines. The intramuscular electromyography examination (iEMG), which requires placement of small needles into several muscles to record their electrical activity, is an invasive procedure and is known to cause discomfort for the subject. If a robust electrode contact with the skin is feasible, electrical activities of shallow muscles can be, to some extent, captured via the non-invasive and convenient method of surface EMG (sEMG). However, sEMG suffers from a higher rate of crosstalk, i.e., misleading signals coming from adjacent muscles (Farina et al., 2004). Clearly, not every muscle is accessible for neither sEMG nor iEMG examination. Moreover, there are many concerns regarding the applicability, reliability, sensitivity, and reproducibility of EMG measurements (Vigotsky et al., 2018). Different segments of the same muscle do not generate consistent electrical signals (Ahamed et al., 2014), and the relationship between the recorded EMG signals and the generated muscle forces is deemed complex (Al Harrach et al., 2017). Due to the safety, ethical, and technical limitations of *in vivo* studies and limited accessibility to deep muscles and peripheral neurons, muscle dynamics cannot be directly measured on live human subjects. Therefore, the estimation of muscle activations and the resultant forces is a longstanding and active area of research. Computational biomechanics is considered, to a limited extent, as one of the few possibilities to understand the neural and muscular activation patterns of humans (Erdemir et al., 2007).

Building controllers for musculoskeletal systems is a challenging task as they are inherently underdetermined due to the disparity between the degrees of freedom of the rigid bodies and the number of skeletal muscles (Lee et al., 2014). The masticatory system is also shown, in theory, to be mechanically redundant; therefore, multiple muscle activation patterns can generate similar motion trajectories and bite forces (Osborn, 1996). This redundancy often results in non-unique solutions for the inverse dynamics problem. In computational modeling and computer animation, the joint torques and muscle excitations are estimated so that the model follows a given motion trajectory while, possibly, considering external forces. In the prior works, the inverse dynamics challenge has been tackled with numerical solvers which have either a static or dynamic viewpoint to the optimization problem.

In the static approach, the problem is solved for each timestep with the most likely set of activations which drive the model closer to the desired trajectory (Otten, 2003). Static optimization has been a popular choice in biomechanics thanks to its simplicity (Seireg and Arvikar, 1975; Pedersen et al., 1997; Thelen et al., 2003). The low computational costs of static solvers have extended their application to complex three-dimensional many-muscle models (Lee et al., 2014). However, this formulation is sensitive to the given trajectory and often results in non-smooth outcomes. An extension to the static optimization is the forward-dynamics assisted tracking where consecutive steps are collectively considered for temporal consistency. This allows for the inclusion of muscle contraction dynamics as a regularization factor to reduce sensitivity to the input kinematics (Erdemir et al., 2007). Dynamic optimization stands on the other end of the spectrum and considers muscle forces, among other performance criteria, as time-dependent variables. It optimizes an integral cost function to address a subset of the mentioned challenges (Anderson and Pandy, 1999, 2001). Even though inverse dynamic solvers are fairly straightforward, they have certain limitations including the inconsistencies between the measured external forces and the body kinematics (Faber et al., 2018), the need to solve complex differential equations, and many more (Kuo, 1998; Hatze, 2002; Fluit et al., 2014). When it comes to choosing solvers, there is always the trade-off between the accuracy and the computational cost. More importantly, any inverse dynamics solution inherently relies on the availability of motion trajectories as inputs; however, kinematics are not often easy to obtain from human and animal subjects and are susceptible to the sensor noise.

Reinforcement learning (RL) is an adaptive control strategy inspired by behavioral psychology where organisms' actions are encouraged or averted through antecedent stimuli. The RL paradigm is very similar to the habit development processes in the basal ganglia of the brain and it is suggested that understanding the RL-based control strategies is helpful in the analysis of human behavior (Yin and Knowlton, 2006). With the rise of deep learning and its integration into the RL framework, unprecedented solutions for control and decision making problems were introduced (Mnih et al., 2013). A deep reinforcement learning (Deep RL) solution is essentially a well-formulated closed-loop system where high capacity neural networks are trained with the feedback mechanism of rewards to learn relatively complex actuation patterns. RL solutions are shown to scale well to high-dimensional state and action spaces for biomechanical control (Abdi et al., 2019a).

The main challenge when using RL solutions for motor control is to design a training algorithm, without much knowledge of the systems' dynamics, which teaches the agent to carry out complex musculoskeletal tasks and maximize a delayed reward signal. In computer graphics, where models are not muscle driven and their validity is not an issue, RL is used to teach agents to mimic locomotion tasks (Peng et al., 2018). In biomechanics, some RL-based solutions have been introduced for the motor control tasks either via muscle activations (Abdi et al., 2019b) or joint activations (Clegg et al., 2018). In human locomotion, most works have focused on arm movement (Golkhou et al., 2005; Jagodnik et al., 2016) and gait control (Peng et al., 2017; Kidziński et al., 2018; Jiang et al., 2019). Recent interdisciplinary collaborations have helped to bridge the gap between reinforcement learning and motor control in biomechanics using the OpenSim and ArtiSynth modeling environments (Kidziński et al., 2018; Abdi et al., 2019a). These efforts gained more traction after the “learning to run” challenge of NeurIPS 2017 where variants of the policy gradient family of controllers were implemented to generate gait patterns (Kidziński et al., 2018).

In this work, we are demonstrating our deep reinforcement learning approach toward learning the neural excitation patterns of mastication. We implemented the Soft Actor-Critic (SAC) reinforcement learning algorithm with a domain-engineered reward function to teach the RL policy how to move the jaw in its 3D Posselt envelope of motion. To address the underdetermined nature of the system, we encourage the agent to minimize the generated muscle forces to reduce the metabolic energy expenditure. The outcome of the proposed training process is a parametric model that acts as the brain of the biomechanical system. Our contributions are 4-fold. Firstly, we design a physiologically accurate jaw model, based on the works of Sagl et al. (2019b), with a new take on the TMJ modeling suited for the computationally demanding training process of reinforcement learning. Secondly, we demonstrate the feasibility of training a neural network to estimate the efficient excitation patterns to drive the jaw model in its domain of motion. Thirdly, we conduct experiments to show the sensitivity of the model to the coefficients of the reward function. We also demonstrate how the model's neural excitations match the expected physiological patterns during standard jaw movements. Lastly, we introduce an analytical framework consisting of a set of quantitative metrics to assess the functional performance of a given masticatory system and report on the performance of different models.

## 2. Materials and Methods

### 2.1. Data Acquisition

The biomechanical model used in this study is constructed based on the clinical data of a healthy male 30-years-old volunteer at the Medical University of Vienna. A single full-skull CT scan was acquired from the participant in the closed-mouth position to model the bony structures (Siemens Sensation 4). The in-slice resolution of the scan was 0.3 × 0.3 mm with a slice thickness of 0.5 mm. A full-skull 3D MRI scan was also acquired to model the origin and insertion points of the masticatory muscles (Siemens Magnetom Prisma 3T with a 64-channel head coil). A coronal Double Echo Steady State T1-weighted sequence with water excitation was used for image acquisition, covering the maxillofacial region down to the shoulders. The resolution of the MRI scan was 0.3 × 0.3 mm with a slice thickness of 0.5 mm. Further details of the data acquisition process are discussed in a prior publication of our team (Sagl et al., 2019a).

Given the central role that teeth play in the masticatory system, and in order to obtain high-resolution dental surfaces, physical plaster models (dental casts) of the subject's dentition were created with Gypsum Stone IV. The dental casts were then digitized with an optical scanner (Ceramill map 400) with 3D accuracy of smaller than 20 μm. The dental segment of the upper and lower jaws obtained from the CT scan was then replaced with the high-resolution 3D optical scans of the dentitions.

### 2.2. Modeling

We used the open-source mechanical modeling platform, ArtiSynth^1^, to implement the 3D biomechanical model of the subject. The model used in this study is based on the validated model of Sagl et al. (2019b), with some alterations in the TMJs and the occlusal surfaces to lower the computational cost. This model consists of three rigid bodies: jaw, skull, and the hyoid bone. To speed up the training, the model is simplified with the fixed hyoid bone assumption. To make up for the loss in the range of mouth opening, the hyoid bone is moved about 10 mm inferoposteriorly according to the values reported by Muto and Kanazawa (1994). The forward simulation steps are computed with the semi-implicit backward Euler method. For stability reasons, small timesteps of 0.001 s are used by the integrator across experiments.

Teeth are assumed to be rigidly attached to the mandible and the collective of them is referred to as the jaw rigid body with an estimated mass of 200 g (Langenbach and Hannam, 1999). The moment of inertia of the jaw is calculated based on its 3D geometry with the uniform density assumption.

#### 2.2.1. Masticatory Muscles

Muscles are modeled as point-to-point Hill-type springs which provide a practical formulation of the muscle contraction mechanism (Hill, 1953). Considering the challenges with the estimation of tissue's biomechanical properties (Blümel et al., 2012), here, muscles' cross-sectional areas, length-tension function, and velocity-tension function are based on the parameters reported in the literature (Langenbach and Hannam, 1999; Peck et al., 2000; Hannam et al., 2008). Each modeled point-to-point muscle resembles a spring that only applies forces along its main axis. While we acknowledge the over-simplifying assumptions with the Hill-type point-to-point muscles, the designed model competes with other masticatory models in complexity, details, and biomechanically relevant intricacies (Koolstra and van Eijden, 2005; Choy et al., 2017).

Large muscles (e.g., temporalis) and those with multiple heads (e.g., masseter and lateral pterygoid) are modeled with multiple actuators. One exciter is assigned to each point-to-point actuator. An exciter is the counterpart of the motor axons that innervate the skeletal muscle. In response to the neural excitation, the muscle contracts according to the Hill-type model's function along the muscle compartment's longitudinal axis. This process is referred to as the excitation-contraction coupling. All neural excitations are parameterized as the normalized ratios of their maximal activation in the 0 to 1 range.

**Figure 1:**
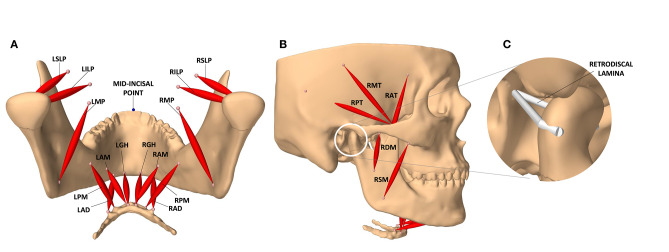
The masticatory model. **(A)** Posterior view of the jaw and the masticatory muscles of the left (L) and right (R) side: anterior belly of digastric (LAD, RAD), posterior mylohyoid fibers (LPM, RPM), anterior mylohyoid fibers (LAM, RAM), geniohyoid (LGH, RGH), medial pterygoid (LMP, RMP), superior head of lateral pterygoid (LSLP, RSLP), and inferior head of lateral pterygoid (LILP, RILP). **(B)** Lateral view of the model and the masticatory muscles: posterior, middle, and anterior fibers of the right temporalis (RPT, RMT, RAT), superficial and deep heads of the right masseter (RSM, RDM). **(C)** The temporomandibular ligaments of the condylar capsule, and other connective tissues contributing to passive retraction of the condyle, are modeled with a multi-point spring which is directly attached to the medial and lateral ends of the condyle.

The modeled masticatory system has 24 actuators and 24 associated exciters (12 on each side) associated with the following muscles: temporalis (3 actuators), lateral pterygoid (2 actuators), masseter (2 actuators), medial pterygoid, anterior digastric, geniohyoid, and mylohyoid (2 actuators on each side). All exciters are assumed to be disjoint, allowing for any excitation pattern to be deemed feasible. The muscle bundles and their exciters are demonstrated in Figures 1A,B.

#### 2.2.2. Temporomandibular Joint

The 3D shape of the condyles and the mandibular fossae (glenoid fossae) are obtained from the CT scan. Given the high computational cost of finite element methods, and in order to speed up the computationally demanding process of reinforcement training, the condylar disks are excluded from the current jaw model. The forward and downward movement of the condyle is guided by a curved bilateral planar constraint mimicking the articular eminence's role during jaw opening. In absence of the condylar disc, and following the original design (Sagl et al., 2019b), the articular cartilage is modeled as an elastic foundation contact model with a thickness of 0.45 mm (Hansson et al., 1977). Based on the available literature, the Young's modulus and Poisson ratio of the elastic foundation are set to 2.7 MPa and 0.49, respectively (Koolstra and van Eijden, 2005). For a fast and stable simulation, the elastic foundation is computed with the constraint regularization method of Servin et al. (2006).

The retrodiscal tissues, superior retrodiscal lamina, and the temporomandibular ligaments of the condylar capsule are modeled with a multi-point spring. This multi-point spring resembles a passive ligament which is wrapped around the condyle and connects its medial and lateral ends to the back of the mandibular fossa and the tympanic plate (Figure 1C). The ligaments grant passive stability to the TMJ. They are designed with a slack length of 7.5 mm longer than the closed jaw position. The Young's modulus of the ligaments is set to 2.45 MPa based on the recent work of Coombs et al. (2017) on the retrodiscal tissue. This design constrains the motion of the condyles inside the mandibular fossae, counters the forward pull of the superior head of the lateral pterygoid, and facilitates the posterior rotation of the condylar neck during jaw closure. It also allows the condyle to slightly reach beyond the summit of the articular eminence at its full stretch during mouth opening which matches that of a healthy subject (Muto et al., 1994).

### 2.3. 3D Envelope of Motion

The 3D envelope of motion was estimated through manual activation of the masticatory muscles in the simulation environment. To calculate the 3D envelope of the current model, a trained dentist used the graphical user interface of the simulation environment to set the excitation levels of the masticatory muscles and drive the jaw to the extremities. The excitations were slowly updated to move the jaw from one end-point to another and the 3D position of the lower mid-incisal point was tracked in between the boundary end-points.

**Figure 2:**
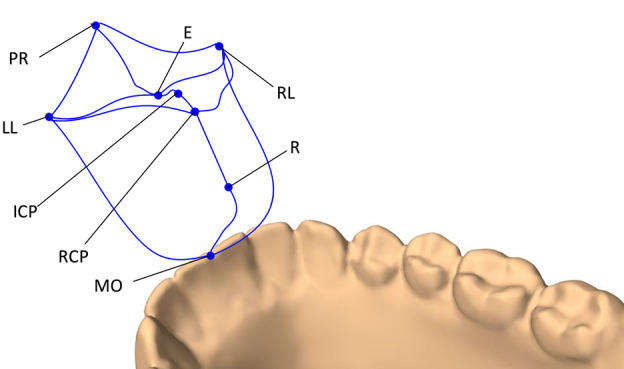
The 3D envelope of motion of the lower mid-incisal point. The end-points of the envelope include: maximum protrusion (PR), edge-to-edge (E), maximum intercuspation position (ICP), retruded contact position (RCP), end of pure rotational opening (R), maximum mouth opening (MO), maximum left laterotrusion (LL), and maximum right laterotrusion (RP).

The end-points of the envelope were decided based on the works of Posselt (1952) and Koolstra et al. (2001). The following jaw positions were used to form the 3D envelope of motion: maximum intercuspal position (ICP), edge-to-edge (E), maximum protrusion (PR), maximum left and right laterotrusion (LL and RL), retruded contact position (RCP), end of the pure rotational opening (R), and maximum mouth opening (MO). The trajectories of the boundary movements of the lower mid-incisal point are visualized in Figure 2.

The 3D envelope of motion is important for this research in three capacities. Firstly, the fact that the simulated 3D envelope of motion resembles that of Posselt's attests the validity of the designed jaw model for the current application. Secondly, it defines the feasible space of motion for the jaw which is necessary for the RL training of the model. Lastly, it defines the optimum motion domain of the non-pathological jaw which acts as a reference for further evaluation of the learned control policies (see section 4).

## 3. Motor Control With Soft Actor-Critic

In reinforcement learning, we often seek to train a policy, π, which maximizes the expected sum of the future rewards (*J*). A policy is simply a mapping from the state space to the action space, i.e., it tells the agent what to do at each situation. This is analogous to the brain's functionality in live subjects. The policy can be a deterministic mapping function or a probabilistic distribution over the possible set of actions. The optimal policy (π^*^) is the one that achieves the maximum rewards and is defined as

(1) $$\begin{array}{l} \pi^{*}=\arg\underset{\pi }{\max}J\left(\pi \right)\;, \end{array}$$

(2) $$\begin{array}{l} J\left(\pi \right)={\text{E}}_{a_{t}\sim \pi \left(.|s_{t}\right)}[\underset{t}{\sum }\gamma^{t}r\left(s_{t},a_{t})\right]\;, \end{array}$$

where *a*_*t*_ and *s*_*t*_ denote the action and the state at time *t*, respectively. In the jaw model, actions are the changes in the neural excitations. Here, γ is the factor that discounts the value of future rewards, i.e., near future rewards are worth more than far future rewards. As mentioned earlier, *J*(π) is the expected sum of future rewards for the policy π, and *r*(.) is the reward function that determines the bonus or penalty associated with actions and state-transitions. The reward function to train the motor control of the jaw model is discussed in section 3.2.

The state-transitions (dynamics model), reward function, and the optimum trajectory to the desired goal are all unknown to the agent in the beginning. It is then the agent's responsibility to interact with the environment and gather information. In reinforcement learning, the agent switches between two strategies to learn about its space, namely, exploration and exploitation. When the agent keeps on pursuing what he believes to be the optimum solution, it is exploiting its learned policy. On the other hand, exploration is when the agent decides to try something new which is not in line with its learned policy. An RL algorithm should maintain a balance between its exploration and exploitation strategies for efficient training.

Different RL algorithms have different takes on how to search for the optimum policy. The Soft Actor-Critic (SAC) algorithm belongs to the family of model-free reinforcement learning. It is an off-policy solution that forms a bridge between stochastic policy optimization and deterministic policy gradient algorithms (Haarnoja et al., 2018a,b). Unlike its many alternatives, SAC is considered to be relatively insensitive to its hyper-parameters which makes it an intriguing option for our current biomechanical modeling setting.

The SAC algorithm contains an entropy term to improve the agent's exploration. The agent is rewarded with respect to the entropy (H) of its learned policy which discourages a premature convergence to sub-optimal deterministic policies (Mnih et al., 2016). The agent is rewarded for randomness (higher entropy) which is also a popular phenomenon in nature (Eysenbach and Levine, 2019). The higher entropy results in more exploration of the environment. It also works as a regularizer that stabilizes the training and is shown to accelerate learning. The SAC formulation for the optimal policy can be summarized as

(3) $$\begin{array}{l} \pi^{*}=\arg\underset{\pi }{\max}\;{\text{E}}_{a_{t}\sim \pi \left(.|s_{t}\right)}[\underset{t}{\sum }\gamma^{t}(r\left(s_{t},a_{t}\right)+\alpha H(\pi \left(.|s_{t}))\right], \end{array}$$

(4) $$\begin{array}{l} H\left(\pi \left(.|s_{t}\right)\right)=\text{E}[-\log\pi \left(.|s_{t})\right], \end{array}$$

which is similar to Equation (2), except for the added entropy term. Here, α is the temperature parameter that determines the relative strength of the entropy regularization term.

In our implementation of the deep SAC algorithm, two parametric models are trained simultaneously, each parameterized by a neural network: the policy function (π) and the action-value function (*Q*), parameterized by ϕ and θ, respectively. The policy function is the *actor* (brain) and the action-value function (*Q*) is its *critic* with the entropy *softening* the expectations, hence the name SAC. During training, at each simulation timestep *t*, the actor receives the current state of the environment *s*_*t*_, processes it and takes the action *a*_*t*_ according to its parametric policy π_ϕ_. As a result of this action, the agent will end up in the new state *s*_*t*+1_.

In the biomechanical model of the jaw, the state is formed of the current and the desired orientation of the jaw as well as the excitation levels of all the masticatory muscles. Given the constrained motion of the jaw, its orientation is abstracted as the position of the mid-incisal point. The muscle excitations are normalized to the [0−1] range. During training, the history of interactions with the environment are stored in a random access memory (D), known as the replay buffer. Each sample in the replay buffer, (*s*_*t*_, *a*_*t*_, *s*_*t*+1_, *r*_*t*_), is a tuple of the current state, executed action, next state, and the reward associated with the transition. The buffer allows for a higher sample efficiency as each sample can contribute to the training of the *Q* and π networks multiple times (Lin, 1992; Mnih et al., 2013).

The action-value function, *Q*_θ_(*s*_*t*_, *a*_*t*_), is the expected reward if, at timestep *t*, the agent takes the action *a*_*t*_ at the state *s*_*t*_, and then continues acting according to the learned policy. In other words, the *Q*-function estimates the value of an action at a given state based on its prospective rewards. Due to the co-dependency of neighboring states, the *Q*-function of SAC can be computed recursively with the modified Bellman operator (Lagoudakis and Parr, 2003) as

(5) $$\begin{array}{l} Q_{\theta }\left(s_{t},a_{t}\right)=r\left(s_{t},a_{t}\right)+\gamma {\text{E}}_{s_{t+1}\sim D}{[V}_{\bar{\theta }}\left(s_{t+1})\right]\;, \end{array}$$

(6) $$\begin{array}{l} V_{\bar{\theta }}\left(s_{t}\right)={\text{E}}_{a_{t}\sim \pi_{\phi }\left(.|s_{t}\right)}[Q_{\bar{\theta }}\left(s_{t},a_{t}\right)-\alpha \log\pi \left(a_{t}|s_{t})\right]\;. \end{array}$$

Here, *V*(.) is the regularized (soft) *state-value* function which is simply called the *value* function. The value function is the expected future reward of a state. In the SAC formulation, this is equal to the value of a state and the expected entropy of the state. In the context of masticatory motor control, the value function roughly indicates how likely it is for the agent to reach its desired position and achieve high rewards if it started from the current jaw orientation and followed its learned policy. Substituting *V*(.) into Equation (5) would result in the recursive Bellman equation for the *Q*-function. While it is possible to learn the state-value function separately using a neural network with independent parameters, in our formulation, the value function is estimated based on the *Q*-function defined in Equation (5).

As discussed earlier, all neural networks are trained based on the randomly drawn samples from the replay buffer (D). The parameters of the *Q*-function are updated with the stochastic gradient descent toward minimizing the mean squared error between the estimated *Q* values, calculated by the *Q*_θ_ function as *Q*_θ_(*s*_*t*_, *a*_*t*_), and the *assumed* ground-truth *Q* value. The assumed ground-truth *Q* values are estimated based on the current reward (*r*_*t*_) and the discounted future reward of the next state (γ*V*_θ_(*s*_*t*+1_)). Accordingly, the mean squared error objective function of the *Q*_θ_ network can be summarized as:

(7) $$\begin{array}{l} J(Q_{\theta })=E_{(s_{t},a_{t},r_{t},s_{t+1})\sim D,\;a_{t+1}\sim \pi_{\phi }}\left[(Q_{\theta }(s_{t},a_{t}))\right]\\ \;-\;[r_{t}+\gamma E_{s_{t+1}\sim D}[V_{\bar{\theta }}(s_{t+1})]]{)}^{2}]. \end{array}$$

In Equation (6), and, consequently, in the nested expectation on the right-hand side of Equation (7), the parameters of the networks are denoted as $\bar{\theta }$. This change in notation is to highlight a stabilizing practice where the critic is modeled with two neural networks with the exact same architecture but independent parameters (Mnih et al., 2015). The secondary network, referred to as the target network and denoted as $Q_{\bar{\theta }}$, is the one that is used to calculate the *assumed* ground-truth value of the next state in Equations (6) and (7). The parameters of the target critic network ($Q_{\bar{\theta }}$) are iteratively updated with the exponential moving average of the parameters of the main critic network (*Q*_θ_). This constrains the parameters of the target network to update at a slower pace toward the parameters of the main critic, which has shown to stabilize the training. It also transforms the ill-posed problem of learning the *Q*-function through bootstrapping (learning estimates from estimates) into a supervised learning problem that can be solved via the gradient descent optimization (Lillicrap et al., 2016).

Another enhancement which played a substantial role in the success of the current motor control solution is the double Q-learning (Hasselt, 2010; Van Hasselt et al., 2016). In this approach, two *Q* networks for both of the main and the target critic functions are maintained. When estimating the current *Q* values or the discounted future rewards, the minimum of the outputs of the two *Q* networks is used:

(8) $$\begin{array}{l} Q_{\theta }\left(s_{t},a_{t}\right)=\min\left(Q_{\theta 1}\left(s_{t},a_{t}\right),Q_{\theta 2}\left(s_{t},a_{t}\right)\right)\;. \end{array}$$

This approach prohibits the estimated *Q* values to grow too large and is found to speed up the training and help achieve higher performing policies (Haarnoja et al., 2018a).

As for the optimal policy (Equation 3), the parameters of π_ϕ_ is updated to maximize the expected future return as well as the expected entropy. If the *Q*-function (critic) is assumed to be telling the truth, finding the optimal policy is the same as maximizing $E_{\pi }\left[V_{\bar{\theta }}\left(s\right)\right]$. This can be expanded, based on Equation (6), as follows

(9) $$\begin{array}{l} J\left(\pi_{\phi }\right)={\text{E}}_{a\sim \pi_{\phi },s\sim D}[Q_{\theta }\left(s,a\right)-\alpha \log\pi \left(a|s)\right]. \end{array}$$

The objective is optimized using the stochastic gradient ascent based on the random samples drawn from the replay buffer (D).

### 3.1. Neural Architectures and Space Definitions

**Figure 3:**
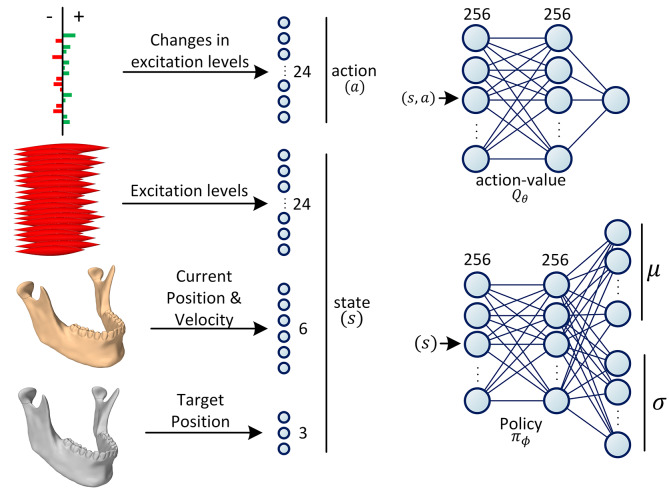
Definition of state and action spaces and the architectures of the neural networks. The action is defined as the increase/decrease in the excitation levels of the muscles. The state is formed of the current and the desired positions of the jaw as well as the current excitation levels of all the masticatory muscles. During training, the policy network (π_ϕ_) predicts the parameters of the Gaussian distribution, N(μ,σ). The action is then randomly sampled from this distribution with the reparameterization trick. During testing, the action is deterministically chosen as the mode of the distribution. During training, the action-value network (*Q*_θ_) predicts the value of a given action-state tuple (*s, a*), which form the components of the objective function (see Equations 7 and 9).

The two functions, *Q*_θ_ and π_ϕ_, are parameterized with neural networks. In all our experiments, the *Q*-network is designed as a 3-layer fully-connected (dense) neural architecture (multi-layer perceptron with two hidden layers) with Rectifier Linear Unit (ReLU) activations after the first two layers. As shown in Figure 3, the sizes of the middle (hidden) layers are set to 256. The *Q*_θ_ network estimates the action-value function denoted as *Q*(*s, a*); therefore, its input size is the sum of the dimensionalities of the action space and the state space and its output size is 1.

The jaw model is formed of 24 masticatory muscles, hence, the action size of 24. In this context, an action is a command sent to the simulation environment to increase or decrease the excitations of a subset of muscles. Change in each muscle's excitation level is capped to the maximum 10% of its excitation range. The jaw orientation is abstracted and approximately represented as the 3D position of the mid-incisal point. The state of the RL agent is then defined as the union of the current neural excitations for the muscles (24 values), the orientation (3 values) and velocity (3 values) of the jaw, and its desired orientation (3 values). Therefore, the size of the state space sums up to 33. In our experiments, the velocity of the desired mid-incisal point is always zero, i.e., the model is trained to reach a static target state.

The policy network is a probability density function estimator that predicts the distribution of actions conditioned on the state. The policy network is formed of two linear hidden layers of size 256, each followed by a ReLU activation function. In our design, we are assuming the policy to have a Gaussian distribution. Therefore, the last layer of the policy network is formed of two parallel linear layers which encode the mean and standard deviation of the N(μ,σ) distribution, respectively (Figure 3). During training, the estimated distribution is sampled with the reparameterization trick for the sampling to be differentiable (Kingma and Welling, 2014). During inference (testing), the mode of the distribution is used for the optimal action selection.

### 3.2. Reward Function

At each timestep, the agent receives a reward value from the environment based on the executed action and its state transition from *s*_*t*_ to *s*_*t*+1_. The success of an RL experiment heavily relies on the design of the reward function. Reward functions are counterparts to the objective functions (loss functions) in the optimization domain; however, in the realm of RL, the agents are trained to maximize the expectation of future rewards. The reward function used in this study is formed of three terms, each of which encourages the agent to pursue a certain goal. If the current position of the mid-incisal point is denoted as *P*_*t*_, and its desired position as $\hat{P}$, the designed reward function can be formulated as:

(10) $$\begin{array}{l} \left(s_{t},a_{t})=-w_{u}\log({\left\|\hat{P}P_{t+1}\right\|}_{2}+\epsilon \right)\\ \;-w_{r}{\left\|f_{t+1}\right\|}_{2}\\ \;-w_{s}{\left\|e^{l}{\;}_{t+1}-e^{r}{\;}_{t+1}\right\|}_{1}, \end{array}$$

where ${\left\|\hat{P}P_{t+1}\right\|}_{2}$ is the second norm of the vector, i.e., the Euclidean distance (in millimeters) of the current mid-incisal point to its desired location. The first term incentivizes the policy (brain) to drive the jaw toward its desired location. The logarithmic nature of this term mitigates the destructive growth of the penalty when the jaw is far away from its deemed target. On the other hand, when the jaw gets to the sub-millimeter distance of the target, the agent is highly positively rewarded. The ϵ value is to avoid the infinite reward in cases where *P* exactly resides at $\hat{P}$. In the second term, ***f*** is the vector of the muscle tensions. The second term acts as a regularizer which minimizes the energy expenditure of the collective of the masticatory muscles. In the third term, ***e*^*l*^** and ***e*^*r*^** are the neural excitations of the left and right side muscles, respectively. Accordingly, the third term calculates the first norm of the differences between the bilateral muscle pairs; thus, it is the non-symmetric penalty that punishes the action if the bilateral muscle pairs are not similarly activated. Finally, *w*_*u*_, *w*_*r*_, and *w*_*s*_ are coefficients that determine the relative weights of the target reaching, the force regularization, and the symmetry terms.

### 3.3. Training Details

The training process is partitioned into independent episodes. At each episode of training, the desired position of the mid-incisal point is randomly chosen from within the 3D envelope of motion (see section 2.3). The random target positioning alternates between two approaches. In 50% of the episodes, the target's position is set as the weighted linear combination of all of the envelope's end-points (Figure 2). The weights of this linear equation are randomly drawn for each episode. In the other 50% of episodes, two of the eight envelope end-points are randomly selected with replacement and the target's position is set as a random linear combination of the two points. This strategy asserts that the boundaries of the envelope are included in the training for the agent to learn the entire domain of motion. Moreover, since the end-point selection is done with replacement, in 0.5 × 0.125 = 6.25% of the episodes the desired jaw position is one of the end-points with the same point chosen in the random selection.

The training can continue for tens of thousands of episodes until no further improvement is noticed in the optimization of the objective functions. An episode runs for a maximum of *T* steps during which the agent interacts with the environment according to its learned policy. An episode ends with the jaw reaching the 100 μm proximity of the desired position or with the agent running out of its maximum *T* allowed steps for the episode.

The parameters of the neural networks are primarily initiated to random values based on the Xavier initialization function (Glorot and Bengio, 2010). Consequently, the agent will start by randomly exploring the space. As the agent interacts with the environment, it collects experiences that eventually get stored in the replay buffer. The weights of *Q*_θ_ and the π_ϕ_ functions are updated at each timestep based on a batch of 256 samples randomly drawn from the replay buffer. As a result, the algorithm alternates between experiencing (filling the replay buffer) and updating the parameters of the *Q*_θ_ and π_ϕ_ networks based on the randomly drawn buffer samples. The parameters of the networks are updated to minimize their respective objective functions (Equations 7 and 9). At the end of each timestep, the parameters of the target critic network, $Q_{\hat{\theta }}$, are updated as the exponential moving average of the parameters of the *Q*_θ_ network based on the target smoothing coefficient τ.

In all of the experiments, the learning rate starts at 0.001 and gradually decreased to 0.0004 at an exponential decay rate of 0.999995. The α value of the SAC algorithm and the reward discount value (γ) are consistently set to 0.3 and 0.99, respectively. The capacity of the replay buffer is generously set to include 1 million samples. The coefficient of the target reaching term, *w*_*u*_, is not updated in between experiments, instead *w*_*r*_ and *w*_*s*_ are tuned. The target smoothing coefficient (τ) is set to 0.005. The maximum number of steps in an episode (*T*) was set to 100.

The training is continued until the objective functions of the actor and the critic converge to a steady-state and stop improving. In our experiments, and with the non-distributed implementation of the training procedure, it took an RL model a few days up to a week to converge. During this time period, the agent went through 10–30k episodes of training, equal to 2–4 million interactions with the environment.

The SAC learning algorithm and the training procedure were implemented in Python and used the ArtiSynth-RL plugin to interact with the ArtiSynth modeling environment (Abdi et al., 2019a). Our implementations of the jaw model and the training algorithm along with the scripts to reproduce the experiments are open-sourced at https://github.com/amir-abdi/artisynth-rl.

## 4. Performance Analysis of Mastication

The performance of mastication can be quantified based on the chewing rhythm, velocity, range of mandible displacements, and the masticatory forces (Xu et al., 2008). The chewing process can be divided into cyclic jaw movements or gape cycles which can be measured through lateral and vertical tracking of the jaw (Laird et al., 2020). According to the literature, a high performing masticatory system is one with a high frequency of cycles, high velocity of mandibular movements, high maximum bite force, and potential for large mandibular displacements. These quantities can be measured with respect to some reference points, such as the lower mid-incisor point and the condylar centers (Ow et al., 1998; Tsuruta et al., 2002). Many studies have focused on the patterns of occlusion and chewing cycle excursions with different bolus types (Anderson et al., 2002; Peyron et al., 2002; Foster et al., 2006). The EMG measurements of muscular activities, albeit variable between subjects, are also shown to have sufficient correlation within a subject across experiments and have been suggested as an efficiency metric (Tortopidis et al., 1998).

The masticatory cycle can be simplified as a tear-drop movement where the mid-incisal point moves downward, then laterally toward the working side, and finally retracted medially to crush the bolus (Murray, 2016). Considering these masticatory movements, we propose a framework for masticatory performance evaluation. This framework is designed to quantify the performance of a given masticatory system based on the following criteria.

### 4.1. Range of Motion (ROM)

To quantify the range of mandibular motion, the boundary envelope of the motion is approximated as a convex space and the volume of this assumed convex hull is calculated. The range of motion is then defined as the percentage of the feasible space achieved by the model. We rely on the reference (optimum) convex hull calculated in section 2.3 by setting the neural excitations.

### 4.2. Metabolic Efficiency (ME)

We are assuming a linear relationship between the muscle tensions and the amount of energy consumed by the muscle fibers. Accordingly, the metabolic efficiency is defined as the inverse of the average muscle tensions during a predefined set of masticatory movements, as follows

(11) $$\begin{array}{l} \text{ME}=(\frac{1}{T}\overset{T}{\underset{t}{\sum }}\bar{f_{t})}{\;}^{-1}\;, \end{array}$$

where *T* is the total number of steps in an episode, and $\bar{f_{t}}$ is the mean of the muscle tension vector at timestep *t*.

### 4.3. Agility (Ag)

Agility is defined as the inverse of the time it takes for the masticatory system to translate the jaw in between a predefined set of locations in the 3D space.

### 4.4. Accuracy (Ac)

The ability of the trained RL policy in driving the body of the mandible toward the desired position is counted as the accuracy of the system. This metric is evaluated as the inverse of the Euclidean distance of the lower mid-incisal point to its desired target position, averaged over multiple episodes, and formulated as

(12) $$\begin{array}{l} \text{Ac}={\left\|\hat{P}P_{T}\right\|}_{2}^{-1}\;, \end{array}$$

where *P*_*T*_ is the location of the lower mid-incisal point after the very last iteration of the episode and $\hat{P}$ is its desired location.

### 4.5. Symmetry (Sym)

The biomechanical system in question is not fully symmetric; however, the current metric is designed to evaluate the extent of symmetric behavior in the trained RL agent. In this context, symmetry is defined as the inverse of the first norm of absolute differences between corresponding excitations of the left and right muscles, averaged over multiple episodes:

(13) $$\begin{array}{l} \text{Sym}=(\frac{1}{T}\sum_{t}^{T}{\left\|\;e^{l}{\;}_{t}-\;e^{r}{\;}_{t}\right\|}_{1}{)}^{-1}\;. \end{array}$$

## 5. Experiments and Results

After the reinforcement learning model is trained, the trained stochastic policy is queried with a state (as defined in Figure 3) and the agent executes the action associated with the mode of the returned distribution. Accordingly, the inference process is deterministic.

### 5.1. Force Regularization

In the first set of experiments, we investigate the impact of the regularization term in the reward function (Equation 10) in the metabolic efficiency of the trained agent. Here, we keep the *w*_*u*_ coefficient consistent across experiments and evaluate the converged model with respect to the *w*_*r*_ coefficient based on the performance metrics discussed in section 4. The non-symmetric coefficient, *w*_*s*_ was set to zero during these experiments.

To test the trained model, a series of 21 target positions on the border and inside the envelope of movement were defined. The agent was then tested based on its ability to navigate the jaw model in between the predefined target locations. The position of the jaw, neural excitations, and muscle forces were tracked throughout the experiments.

**Figure 4:**
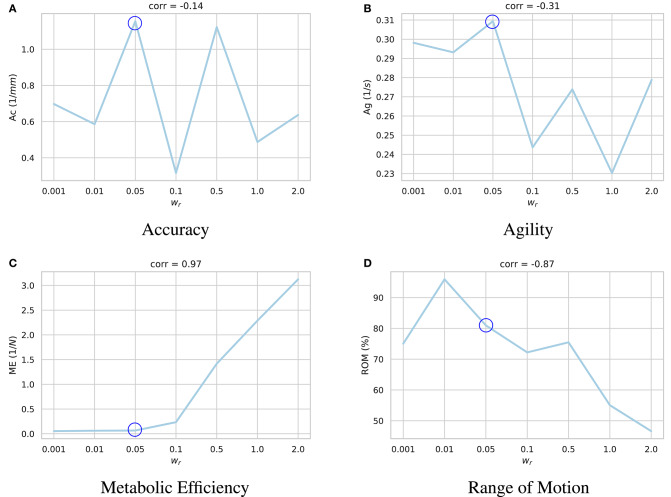
Impact of the regularization coefficient (*w*_*r*_) on performance of the learned motor control based on **(A)** accuracy, **(B)** agility, **(C)** metabolic efficiency, and **(D)** range of motion. Given the under-determined nature of the masticatory system, it is expected of the reinforcement learning policies to act differently according to their respective reward functions and the randomness in their training process. For each sub-figure, seven models were independently trained with different *w*_*r*_ parameters and their performances were quantified. As demonstrated, while increasing *w*_*r*_ increases the metabolic efficiency (ME) of the experiment with a high correlation of 0.97, it has a degrading impact on the model's range of motion (ROM). Force regularization was shown to have no impact on the model's accuracy. The model's agility decreased on average in highly regularized models. As shown here, the model trained with *w*_*r*_ = 0.5 (circled in all sub-plots) demonstrates a balanced performance across the metrics.

The results of these experiments are summarized in Figure 4. As demonstrated, higher values of the regularization coefficient result in more metabolically efficient muscle activation trajectories according to the model's perception of metabolic efficiency, i.e., the muscular tensions (section 3.2). However, with the model thriving for the least amount of applied force, its agility slightly decays. For example, in highly regularized models, the agent delegates the responsibility of jaw elevation to the passive ligaments of the TMJ. Accordingly, mouth closing is carried out at a slower pace (see Supplementary Video 1).

A high negative correlation (ρ = −0.87) was also observed between *w*_*r*_ and the model's range of motion. It is our understanding that with an excessive force regularization, lowering the metabolic energy expenditure becomes a priority as the agent is highly penalized to activate its muscles. Since reaching the edges of the envelope of motion requires higher muscle activations, the agent decides not to reach the edges to save more energy.

### 5.2. Symmetric Behavior

Similar to live subjects, the masticatory model designed in ArtiSynth is not completely symmetric. Consequently, the RL agent as well does not learn symmetric excitation patterns for the left and right muscles. To explore the impact of the symmetry term of the reward function (Equation 10) in the RL training process and to understand the neuromuscular activation patterns, a set of models were trained with different *w*_*s*_ coefficient values. The models were trained with *w*_*r*_ = 0.5 as a balanced solution between speed, accuracy, range of motion, and the least neural excitations, based on the results presented in section 5.1. Given that the neural excitations have a value between 0 and 1, larger *w*_*s*_ values were used to incentivize symmetric activations compared to the *w*_*r*_ values. The values of the *w*_*u*_ and *w*_*r*_ coefficients were kept consistent in these sets of experiments.

**Figure 5:**
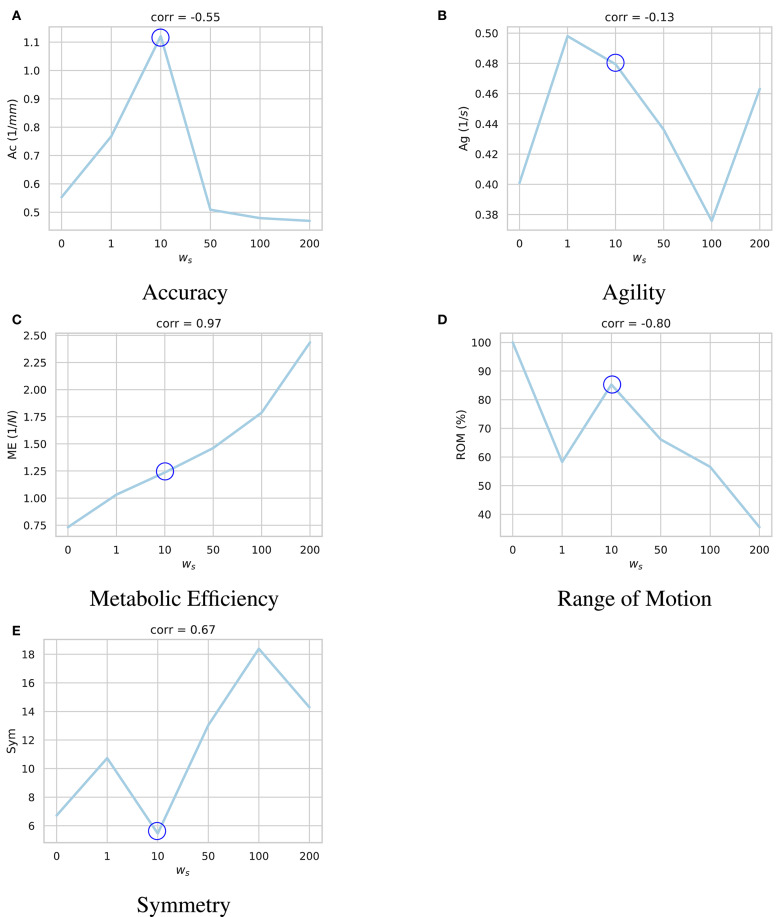
Impact of the symmetry coefficient (*w*_*s*_) on the learned motor control based on **(A)** accuracy, **(B)** agility, **(C)** metabolic efficiency, **(D)** range of motion, and **(E)** symmetry. For each sub-figure, six models were independently trained with different *w*_*s*_ parameters and their performances were quantified. As shown, bigger *w*_*s*_ coefficient incentivizes the agent to learn more symmetric activation patterns. However, such model is less accurate, and is not able to explore as much of the state space. Moreover, the non-symmetric penalty (Equation 10) has a regularization effect which decreases the overall neural excitations and increases the metabolic efficiency (ME). Accordingly, the model trained with *w*_*s*_ = 10 (circled in all sub-plots) demonstrates a balanced performance across the metrics.

The same series of target positions as the previous section were chosen and the trained models were tested based on the same performance criteria. As plotted in Figure 5, higher *w*_*s*_ values encourage the model to learn symmetric activation patterns for the left and right muscles. However, with more symmetric behavior, the model becomes damper and fails to explore its feasible domain of motion. Moreover, the non-symmetric penalty is shown to have a regularization effect, similar to the force regularization, which decreases the overall neural excitations and increases the metabolic efficiency (ME) of the model.

### 5.3. Muscle Activation Patterns

To understand the learned dynamic patterns, a trained agent was set to navigate in the 3D space in between a predefined series of positions. The neural excitation patterns were recorded during these movement. Here, we used the model trained with *w*_*r*_ = 0.5 and *w*_*s*_ = 10 as it demonstrated the most balanced performance.

**Figure 6:**
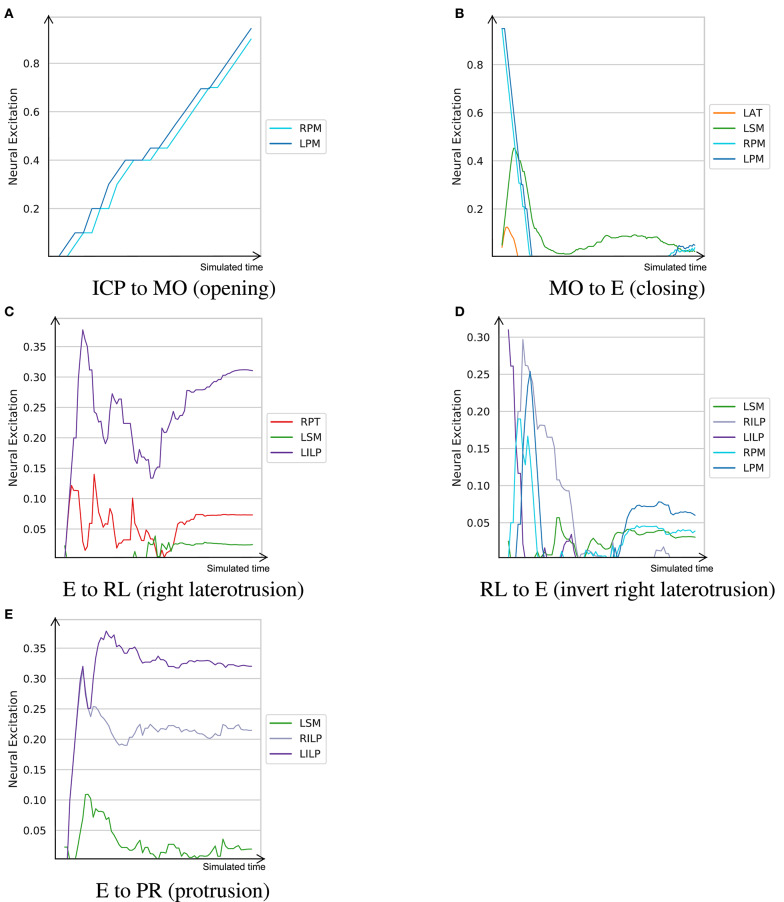
The learned neural excitation patterns for typical masticatory functions. Intercuspal position (ICP) to maximum mouth opening (MO) demonstrates the mouth opening function. Mouth closing is denoted as MO to E. Edge-to-edge position (E) to right laterotrusive (RL) simulates the right laterotrusion function, and RL to E is its opposite. The protrusive function is denoted as E to PR. **(A)** ICP to MO (opening). **(B)** MO to E (closing). **(C)** E to RL (right laterotrusion). **(D)** RL to E (invert right laterotrusion). **(E)** E to PR (protrusion).

The neural excitation trajectories recorded in this experiment are visualized in Figure 6. As demonstrated, while some of the observations do not match perfectly with our expectations, they follow our knowledge of the masticatory system to a good extent. For example, in the right laterotrusion function (E to RL), the contralateral (left side) lateral pterygoid muscle (LILP) does the heavy lifting, assisted by the ipsilateral posterior temporalis muscle and slightly by the contralateral superficial masseter (Bakke, 2016). To return the jaw to the edge-to-edge position (RL to E), the left lateral pterygoid abruptly relaxes and the right lateral pterygoid (RILP) takes over. When the jaw arrives at the mid-sagittal position, RILP relaxes at a slower pace. This process is assisted by the ipsilateral masseter; consequently, to counterbalance the closing force of the left-side masseter, the posterior fibers of the right mylohyoid (RPM) are slightly (close to 3%) activated to keep the jaw in the edge-to-edge position. Similarly, the protrusive movement of the mandible (E to PR in Figure 6) is driven by the bilateral contraction of the lateral pterygoid muscles (Ho, 2017).

Since smaller muscles tend to produce less force, and in turn, consume less energy, a metabolically efficient model (such as one trained with *w*_*r*_ = 0.5) prioritizes activation of small muscles over large ones for certain tasks. From the visualized experiments, the mouth opening task (ICP to MO) is carried out by activating the bilateral posterior mylohyoid fibers, while other muscles, such as the anterior digastric and inferior head of the lateral pterygoid, remain inactive. While this observation is not in sync with our understanding of the masticatory system, the model's decision is optimal according to its own understanding of the metabolic efficiency. This experiment was conducted to highlight that the proposed approach to learning neural excitation patterns is a good tool for generating hypotheses. However, without subject-specific models and rigorous studies of the reward functions and fine-tuning of the learning coefficients, it is not possible to establish a learning paradigm that mimics the human neural system.

Another example is jaw elevation (MO to E) where the mylohyoid muscles suddenly relax and the closing muscles, namely the temporalis and masseter, are activated to bring the condyle back to the glenoid fossa. The passive force of the condylar ligaments, modeled with the multi-point springs, play an important role in the jaw closure; therefore, once the translational phase of jaw closure is over, all muscles temporarily relax and then get slightly activated to establish the edge-to-edge relationship. Accordingly, the agent exploits the passive force of the condylar ligaments to minimize its energy expenditure during mouth closing.

Supplementary Video 1 captures the dynamics of the jaw during the experiments described in this section.

## 6. Discussion

We present a reinforcement learning (RL) approach to estimate the neural excitations of the masticatory muscles. The implemented RL algorithm in this research is based on the Soft Actor-Critic (SAC) formulation which promotes policies with higher entropies. The SAC has demonstrated results that outperform other off-policy and on-policy state-of-the-art RL algorithms, such as the Deep Deterministic Policy Gradient (Lillicrap et al., 2016), Trust Region Policy Optimization (Schulman et al., 2015), and the Proximal Policy Optimization (Schulman et al., 2017), both in terms of performance and sample efficiency (Haarnoja et al., 2018a). In our design, the actor and critic functions of SAC were parameterized by relatively shallow neural networks. We also leveraged double Q-learning and used a separate target critic network to stabilize the learning process.

As demonstrated in section 5.3, the muscle excitation trajectories during opening, closing, laterotrusive, and protrusive movements matches the known physiological patterns (Figure 6). Accordingly, the left and right pterygoid muscles play a substantial role in laterotrusive and protrusive movements. We would like to highlight that, similar to live subjects, the jaw model in question is not symmetric. This asymmetry is apparent in muscle insertion sites, the location of the curved bilateral planar constraints for the TMJ, and the shapes of the teeth. Accordingly, it is not expected to observe symmetric excitation patterns especially when the RL policy is mainly opting to minimize the metabolic cost of its actions. As a result, and as Figure 6 represents, muscles of the left side are more predominantly activated even during symmetric movements, such as jaw closing and protrusion. The non-symmetry penalty term in the reward function mitigates this issue to some extent (Figure 5); however, this gain in symmetry comes with the cost of a lower range of motion. A smarter reward function is deemed necessary to achieve higher performing results.

There have been quite a few metabolic analyses of gait that consider ground reaction forces, motion trajectories of limbs, and pulmonary gas exchanges to estimate the metabolic cost of walking and running. Based on these metrics, multiple metabolic models are introduced which demonstrate consistent results in estimating the relative metabolic cost of different gait tasks (Koelewijn et al., 2019). However, the question of *what is being optimized in biomechanical systems during mechanical tasks* remains unanswered. Should efficiency be defined as the minimum metabolic cost or should the mechanical work be also included as a second indicator of efficient movements (Fetters and Holt, 1990)? Moreover, whether the fidelity requirements of the task play a role in the metabolic efficiency trade-off requires further investigations. In the neural excitation trajectories presented in Figure 6, small muscles seem to get activated more often while large muscles are seldomly activated and merely used for balancing. This is certainly the case for mouth opening where the entire task is handled by the posterior fibers of the left and right mylohyoid muscles.

As discussed earlier, motor control of biomechanical systems is often an underdetermined problem as there are more muscles than the collective of degrees of freedom of the bodies (Lee et al., 2014). Consequently, the local maxima that the model's policy in the RL training framework converges to is not unique either. From the trained agent's perspective, at any state, there is a distribution of actions to choose from. The agent can be instructed to act based on either the mode of the distribution or a randomly drawn sample from the distribution. Regardless, an infinite number of policies can be trained, differing in their respective reward functions and other aspects of their RL formulation, each of which could converge to a different, but to some extent justified, local maxima. Both the force regularization and symmetric terms of the reward function (Equation 10) constrain the solution space of the model; however, these constraints are rather soft and do not guarantee a unique solution.

The RL training procedure is computationally intensive and can take a few hours to a few days depending on the training algorithm, hardware, the parallel efficiency of the implementation, and the dimensionality of the action and the space states. However, once the policy is trained, it is faster than any inverse dynamics solvers as it does not require an iterative numerical method to estimate the excitation and forces at every simulation time step (Abdi et al., 2019a). In deep RL, a trained policy is a feed-forward or recurrent neural network which has learned a deterministic or probabilistic mapping between the state space and the action space. Therefore, at a given state, the action can be inferred via a single feed-forward passing of the model which is quite fast for the shallow neural architectures used in the deep RL paradigm. In our experiments, the feed-forward pass of the policy network (see π_ϕ_ in Figure 3) took <1 ms running on a GeForce GTX 1080. This is one-tenth of the time needed for a single forward step of the jaw simulation in ArtiSynth, running on an Intel Core i7-8700K 3.70 GHz processor, without even taking into account the overhead of the iterative inverse dynamics solver.

Similar to other machine learning and RL settings, the hyper-parameter space was deemed bigger than what could be fully investigated with our limited computational resources. However, within the limitations of our study, we found the entropy coefficient (α in Equation 9) to play a substantial role in the rate of convergence. According to our findings, and due to the relatively high dimensional action space, an α value of <0.2 does not incentivize the agent to explore the environment with an adequate frequency which, in turn, slows down the learning process.

In a simulated mechanical system, it is possible to assess the feasibility of a hypothetical motion trajectory by estimating the motor control resulting in the given kinematics via forward or inverse dynamics solutions. Therefore, the proposed RL training framework along with the masticatory performance metrics can be a viable solution to predict the post-operative functionalities of a subject. Patients who come out of extensive jaw reconstructive surgeries suffer from impaired masticatory functions. The brain of a post-operative patient, who has just come to realize its altered masticatory system, is in the self-experimental phase, meaning that it interacts with the environment through experiments and makes predictions on the results of those interactions to decide the best trajectory. As the patient fails to predict the results of the sensorimotor predictions, it enters a self-repairing phase where it starts to adapt to new world dynamics and compensate for lost motor abilities by finding new paths and activation patterns. This brain is also self-growing as it rebuilds the dynamics model of the jaw via gathering new information through experiments (Corbacho, 2016). The reinforcement learning process designed in this study is in sync with the three qualities of the post-operative brain, namely, self-experience, self-repair, and self-grow. While the subject is going through rehabilitation, the clinical team is curious to know, in theory, the extent to which the subject is expected to regain masticatory performance. With the proposed approach, such questions can be answered through virtual surgical interventions in the simulation platform and retraining of the RL agent to evaluate its adaptation with the new environment. Moreover, if multiple surgical avenues are available at the time of treatment planning, the surgeon and, in turn, the patient would benefit from knowing if an alternative plan could result in a more optimal functional outcome. We see a future where the proposed training framework is coupled with subject-specific biomechanical models as a benchmarking platform and to answer the *what-if* questions which are often raised during treatment planning of surgeries.

## 7. Limitations and Future Work

Although the main contribution of this work is not to present the most accurate and validated subject-specific jaw model, we acknowledge the simplifying assumptions made in the biomechanical modeling as well as the policy training procedure. We would like to elaborate on some of these limitations to highlight potential avenues for enhancement. We divide these details into two categories, namely, limitations of the biomechanical model and those of the reinforcement learning procedure.

In order to speed up the costly process of the RL training, no component was modeled as finite elements. Consequently, and in contrast to previous works of Sagl et al. (2019b), the condylar disks and other soft tissues associated with the stomatognathic system were excluded from the model. While we acknowledge that the condylar disks play a non-trivial role in the masticatory system, we compensated for the loss of accuracy by modeling the condylar capsule and the adjacent tissues as passive multi-point springs. The cartilages were also modeled with elastic foundations (Servin et al., 2006). Moreover, and similar to all the jaw models presented in the literature, teeth were assumed to be rigidly connected to the jaw bone with no periodontal ligaments (PDL). In a more favorable setting with an abundance of computational power and time, each tooth can be modeled separately with a PDL that enables a limited extent of physiological movement.

In the designed stomatognathic system, the hyoid bone was assumed to be static and was moved inferoposteriorly to compensate for the loss in the amount of jaw opening. Moreover, some ligaments which contribute to the passive retraction of the jaw were not modeled, such as the stylomandibular ligament and the sphenomandibular ligament.

Here, muscles were modeled as point-to-point axial springs and large muscles were represented with multiple actuators. This simplifying assumption matches the conventional wisdom of prior arts, yet is not in sync with reality. Moreover, the muscles are assumed homogeneous along their length and their force functions follow the well-known Hill-type model. As a result, the muscles ignore the impact of temporal dependency and the recent contractile conditions of the muscle on their generated force (Arslan et al., 2019).

Along the same lines, in the current formulation, the muscle activations were not delicately modeled. Firstly, no delays were considered between the neural stimulation and muscle contraction. Encoding such delay is deemed complex given the discrete temporal simulation with fixed timesteps of 0.001 s. Moreover, a monotonically increasing mapping was assumed between the excitation level and the muscle activation, i.e., higher neural excitation would always result in a higher muscular tension.

Last but not least, we would like to highlight that, similar to other biomechanical models, the current jaw model relies on the parameters reported in the literature, such as the mass of jaw, tissue materials, force-length properties of the muscles, properties of the condylar capsule, and many more. We cannot imagine this issue to be resolved in the near future with the current imaging and sensing technologies. This limitation has an adverse impact on the validity of any subject-specific models designed for treatment planning. Considering the open-source nature of our research, we invite other researchers to join hands in improving upon the model by including more details and alleviating the above limitations.

As for the limitations of the learning procedure, we should highlight the sample inefficiency of model-free approaches, such as the Soft Actor-Critic algorithm. Although the implemented process leverages a replay buffer memory which holds 1 million samples, it is our understanding that the current training process does not efficiently use the gathered samples. In our implementation, after each environment interaction, a batch of 256 samples are drawn from the replay buffer to apply a single update on the weights of the actor and critic networks. Therefore, a viable next-step would be to dynamically adjust the number of parameter updates per environment interaction over time to achieve a faster and more sample-efficient training. Another enhancement is to change the paradigm toward model-based or hybrid reinforcement learning solutions (Nagabandi et al., 2018). Given the stationary state of the masticatory simulation environment, model-based approaches are expected to achieve the same performance with fewer environment interactions.

In the designed motor control paradigm, the neural pathways are assumed to be completely disjoint. This enables the reinforcement learning actor to activate each muscle independently. This assumption is not well-aligned with the reality of biomechanical systems and the reality of muscular synergies where co-activation of muscles that share the descending or afferent neural pathways produce the kinematic trajectories (Bizzi and Cheung, 2013). In a similar work by Ruckert and d'Avella (2013), a movement primitive representation was proposed which employed parameterized basis functions to exploit the hypothesized muscle co-activations. Accordingly, the shared knowledge between muscles simplified the policy search in high-dimensional action spaces. Coupling the neural excitations limits the degrees of freedom and decreases the dimensionality of action space; thus, it results in a more efficient exploration of the smaller action space which makes the job of the RL agent easier. On the other hand, it raises the concern of the *correctness* of neural couplings. Since Kutch and Valero-Cuevas (2012) have debated the assumption of same neural origins for the muscle synergies and argued that constraints arising from the biomechanics could also result in certain couplings across the muscles, we kept the neural pathways independent in this research. However, this avenue needs further exploration.

An important unanswered question in the training is centered around the reward function. As shown in Figure 4, different muscle force regularization coefficients would result in substantially different policies (brains). One policy could be more agile while the other one generates the least amount of muscle tensions. Finding the right balance between the agent's incentives and validating the outcomes with *in vivo* studies or against the available literature will be a valuable and enlightening research project.

Lastly, a fascinating next step would be to include the occlusal forces in the process and design the reward function for the agent to learn a complete chewing cycle. Such rewarding mechanism should be taking the masticatory rhythm and interocclusal forces into account. However, some version of bolus modeling might be necessary to achieve reliable results.

## 8. Conclusion

In this work, we present a new perspective into estimating the neural excitations of the masticatory musculoskeletal system based on the paradigm of reinforcement learning. In this approach, an RL agent is trained to drive the mandible across the 3D envelope of motion in the simulation environment. The proposed method does not require dynamic clinical measurements, such as EMG, kinematics, or joint force trajectories; instead, the model explores the feasible domain of motion via environment interactions and learns the right excitation patterns from its own experiments. We demonstrate that the agent can be trained to optimize over three objectives: minimizing the distance to the target, maximizing the metabolic efficiency of the movement, and maximizing the symmetric behavior of the left and right neural excitations. The trained models demonstrate excitation trajectories that match the known physiological patterns. The proposed approach does not rely on the availability of the recorded kinematics, therefore, it is deemed as an intriguing alternative for the inverse dynamics problem.

## Data Availability Statement

The biomechanical model, the reinforcement learning algorithm, and scripts to reproduce the reported results are publicly accessible at https://github.com/amir-abdi/artisynth-rl.

## Ethics Statement

The ethics application for data collection involving human participants were reviewed and approved by the Institutional Review Board of the Medical University of Vienna. The participant provided his written informed consent to participate in this study. Data sharing agreements were signed between the participating institutions, namely the Medical University of Vienna, Austria, and the University of British Columbia, Canada.

## Author Contributions

AA and VS designed the study and the experiments based on comments from IS, BS, and SF. AA, BS, and IS developed the biomechanical model. AA and VS drafted the manuscript with valuable inputs from BS. BS, SF, and IS reviewed and edited the manuscript. PA and EP played a supervisory role in the research, overseeing the progress and commenting on important aspects.

## Conflict of Interest

The authors declare that the research was conducted in the absence of any commercial or financial relationships that could be construed as a potential conflict of interest.

## Footnotes

^1^www.artisynth.org

## Supplementary Material

Supplementary Video 1 Demonstrates some of the muscle activation patterns of the masticatory system learned with the designed reinforcement learning approach.

## References

[B1] Abdi A. H. Malakoutian M. Oxland T. Fels S. (2019a). Reinforcement learning for high-dimensional continuous control in biomechanics: an intro to artisynth-rl, in Deep Reinforcement Learning Workshop, 33rd Conference on Neural Information Processing Systems NeurIPS (Vancouver, BC).

[B2] Abdi A. H. Saha P. Srungarapu V. P. Fels S. (2019b). Muscle excitation estimation in biomechanical simulation using NAF reinforcement learning, in Computational Biomechanics for Medicine, eds M. P. Nash, P. M. F. Nielsen, A. Wittek, K. Miller, and G. R. Joldes (Springer International Publishing), 133–141. 10.1007/978-3-030-15923-8_11

[B3] Ahamed N. U. Sundaraj K. Alqahtani M. Altwijri O. Ali M. A. Islam M. A. (2014). EMG-force relationship during static contraction: effects on sensor placement locations on biceps brachii muscle. Technol. Health Care 22, 505–513. 10.3233/THC-140842 25059255

[B4] Al Harrach M. Carriou V. Boudaoud S. Laforet J. Marin F. (2017). Analysis of the sEMG/force relationship using HD-sEMG technique and data fusion: a simulation study. Comput. Biol. Med. 83, 34–47. 10.1016/j.compbiomed.2017.02.003 28219032

[B5] Anderson F. C. Pandy M. G. (1999). A dynamic optimization solution for vertical jumping in three dimensions. Comput. Methods Biomech. Biomed. Eng. 2, 201–231. 10.1080/10255849908907988 11264828

[B6] Anderson F. C. Pandy M. G. (2001). Dynamic optimization of human walking. J. Biomech. Eng. 123, 381–390. 10.1115/1.1392310 11601721

[B7] Anderson K. Throckmorton G. S. Buschang P. H. Hayasaki H. (2002). The effects of bolus hardness on masticatory kinematics. J. Oral Rehabil. 29, 689–696. 10.1046/j.1365-2842.2002.00862.x 12153460

[B8] Arslan Y. Z. Karabulut D. Ortes F. Popovic M. B. (2019). Exoskeletons, exomusculatures, exosuits: dynamic modeling and simulation, in Biomechatronics (Elsevier), 305–331. 10.1016/B978-0-12-812939-5.00011-2

[B9] Bakke M. (2016). Jaw muscle disorders, in Functional Occlusion in Restorative Dentistry and Prosthodontics, eds I. Klineberg and S. E. Eckert (Elsevier), 173–187. 10.1016/B978-0-7234-3809-0.00014-0

[B10] Bizzi E. Cheung V. C. K. (2013). The neural origin of muscle synergies. Front. Comput. Neurosci. 7:51. 10.3389/fncom.2013.00051 23641212 PMC3638124

[B11] Blümel M. Hooper S. L. Guschlbauerc C. White W. E. Büschges A. (2012). Determining all parameters necessary to build hill-type muscle models from experiments on single muscles. Biol. Cybern. 106, 543–558. 10.1007/s00422-012-0531-5 23132431 PMC3505888

[B12] Choy S. E. M. Lenz J. Schweizerhof K. Schmitter M. Schindler H. J. (2017). Realistic kinetic loading of the jaw system during single chewing cycles: a finite element study. J. Oral Rehabil. 44, 375–384. 10.1111/joor.12501 28258640

[B13] Clegg A. Yu W. Tan J. Liu C. K. Turk G. (2018). Learning to dress. ACM Trans. Graph. 37, 1–10. 10.1145/3272127.3275048

[B14] Coombs M. Petersen J. Wright G. Lu S. Damon B. Yao H. (2017). Structure-function relationships of temporomandibular retrodiscal tissue. J. Dental Res. 96, 647–653. 10.1177/0022034517696458 28530471 PMC5444618

[B15] Corbacho F. J. (2016). Towards the self-constructive brain: emergence of adaptive behavior. arXiv 1608.02229.

[B16] Drake R. Vogl A. W. Mitchell A. (2014). Gray's Anatomy for Students. London: Churchill Livingstone.

[B17] Erdemir A. McLean S. Herzog W. van den Bogert A. J. (2007). Model-based estimation of muscle forces exerted during movements. Clin. Biomech. 22, 131–154. 10.1016/j.clinbiomech.2006.09.005 17070969

[B18] Eysenbach B. Levine S. (2019). If maxent rl is the answer, what is the question? *arXiv* 1910.01913.

[B19] Faber H. van Soest A. J. Kistemaker D. A. (2018). Inverse dynamics of mechanical multibody systems: an improved algorithm that ensures consistency between kinematics and external forces. PLoS ONE 13:e0204575. 10.1371/journal.pone.0204575 30265727 PMC6161892

[B20] Farina D. Merletti R. Indino B. Graven-Nielsen T. (2004). Surface emg crosstalk evaluated from experimental recordings and simulated signals. Methods Inform. Med. 43, 30–35. 10.1055/s-0038-1633419 15026832

[B21] Fetters L. Holt K. (1990). Efficiency of movement: biomechanical and metabolic aspects. Pediatr. Phys. Ther. 2, 155–159. 10.1097/00001577-199002030-00008

[B22] Fluit R. Andersen M. Kolk S. Verdonschot N. Koopman H. (2014). Prediction of ground reaction forces and moments during various activities of daily living. J. Biomech. 47, 2321–2329. 10.1016/j.jbiomech.2014.04.030 24835471

[B23] Foster K. D. Woda A. Peyron M. A. (2006). Effect of texture of plastic and elastic model foods on the parameters of mastication. J. Neurophysiol. 95, 3469–3479. 10.1152/jn.01003.2005 16709719

[B24] Gallo L. M. Fushima K. Palla S. (2000). Mandibular helical axis pathways during mastication. J. Dental Res. 79, 1566–1572. 10.1177/00220345000790080701 11023276

[B25] Glorot X. Bengio Y. (2010). Understanding the difficulty of training deep feedforward neural networks, in Proceedings of the Thirteenth International Conference on Artificial Intelligence and Statistics (Sardinia), 249–256.

[B26] Golkhou V. Parnianpour M. Lucas C. (2005). Neuromuscular control of the point to point and oscillatory movements of a sagittal arm with the actor-critic reinforcement learning method. Comput. Methods Biomech. Biomed. Eng. 8, 103–113. 10.1080/10255840500167952 16154874

[B27] Haarnoja T. Zhou A. Abbeel P. Levine S. (2018a). Soft actor-critic: off-policy maximum entropy deep reinforcement learning with a stochastic actor, in Proceedings of the 35th International Conference on Machine Learning, Volume 80 of Proceedings of Machine Learning Research, eds J. Dy and A. Krause (Stockholm: Stockholmsmässan), 1861–1870.

[B28] Haarnoja T. Zhou A. Hartikainen K. Tucker G. Ha S. Tan J. (2018b). Soft actor-critic algorithms and applications. arXiv 1812.05905.

[B29] Hannam A. Stavness I. Lloyd J. Fels S. (2008). A dynamic model of jaw and hyoid biomechanics during chewing. J. Biomech. 41, 1069–1076. 10.1016/j.jbiomech.2007.12.001 18191864

[B30] Hansson T. Öberg T. Carlsson G. E. Kopp S. (1977). Thickness of the soft tissue layers and the articular disk in the temporomandibular joint. Acta Odontol. Scand. 35, 77–83. 10.3109/00016357709064126 266827

[B31] Hasselt H. V. (2010). Double q-learning, in Advances in Neural Information Processing Systems (Lake Tahoe), 2613–2621.

[B32] Hatze H. (2002). The fundamental problem of myoskeletal inverse dynamics and its implications. J. Biomech. 35, 109–115. 10.1016/S0021-9290(01)00158-0 11747889

[B33] Hill A. V. (1953). The mechanics of active muscle. Proc. R. Soc. Lond. B Biol. Sci. 141, 104–117. 10.1098/rspb.1953.0027 13047276

[B34] Ho S. (2017). Temporomandibular joint, in Orthopaedic Physical Therapy Secrets (Elsevier), 490–494. 10.1016/B978-0-323-28683-1.00061-8

[B35] Jagodnik K. M. Thomas P. S. van den Bogert A. J. Branicky M. S. Kirsch R. F. (2016). Human-like rewards to train a reinforcement learning controller for planar arm movement. IEEE Trans. Hum. Mach. Syst. 46, 723–733. 10.1109/THMS.2016.255863028475063

[B36] Jiang Y. Wouwe T. V. Groote F. D. Liu C. K. (2019). Synthesis of biologically realistic human motion using joint torque actuation. ACM Trans. Graph. 38, 1–12. 10.1145/3306346.3322966

[B37] Kidziński Ł. Mohanty S. P. Ong C. F. Huang Z. Zhou S. Pechenko A. (2018). Learning to run challenge solutions: adapting reinforcement learning methods for neuromusculoskeletal environments, in The NIPS '17 Competition: Building Intelligent Systems, eds S. Escalera and M. Weimer (Cham: Springer International Publishing), 121–153. 10.1007/978-3-319-94042-7_7

[B38] Kingma D. P. Welling M. (2014). Auto-encoding variational bayes, in 2nd International Conference on Learning Representations, ICLR 2014, April 14–16, 2014, Conference Track Proceedings (Banff, AB).

[B39] Koelewijn A. D. Heinrich D. van den Bogert A. J. (2019). Metabolic cost calculations of gait using musculoskeletal energy models, a comparison study. PLoS ONE 14:e0222037. 10.1371/journal.pone.0222037 31532796 PMC6750598

[B40] Koolstra J. Naeije M. Eijden T. V. (2001). The three-dimensional active envelope of jaw border movement and its determinants. J. Dental Res. 80, 1908–1912. 10.1177/00220345010800100901 11706950

[B41] Koolstra J. van Eijden T. (2005). Combined finite-element and rigid-body analysis of human jaw joint dynamics. J. Biomech. 38, 2431–2439. 10.1016/j.jbiomech.2004.10.014 16214491

[B42] Kuo A. D. (1998). A least-squares estimation approach to improving the precision of inverse dynamics computations. J. Biomech. Eng. 120, 148–159. 10.1115/1.2834295 9675694

[B43] Kutch J. J. Valero-Cuevas F. J. (2012). Challenges and new approaches to proving the existence of muscle synergies of neural origin. PLoS Comput. Biol. 8:e1002434. 10.1371/journal.pcbi.1002434 22570602 PMC3342930

[B44] Lagoudakis M. G. Parr R. (2003). Least-squares policy iteration. J. Mach. Learn. Res. 4, 1107–1149. 10.5555/945365.964290

[B45] Laird M. F. Ross C. F. O'Higgins P. (2020). Jaw kinematics and mandibular morphology in humans. J. Hum. Evol. 139:102639. 10.1016/j.jhevol.2019.102639 31841671

[B46] Langenbach G. Hannam A. (1999). The role of passive muscle tensions in a three-dimensional dynamic model of the human jaw. Archiv. Oral Biol. 44, 557–573. 10.1016/S0003-9969(99)00034-5 10414871

[B47] Lee Y. Park M. S. Kwon T. Lee J. (2014). Locomotion control for many-muscle humanoids. ACM Trans. Graph. 33, 1–11. 10.1145/2661229.2661233

[B48] Lillicrap T. P. Hunt J. J. Pritzel A. Heess N. Erez T. Tassa Y. (2016). Continuous control with deep reinforcement learning, in International Conference on Learning Representations (ICLR) (San Juan).

[B49] Lin L.-J. (1992). Self-improving reactive agents based on reinforcement learning, planning and teaching. Mach. Learn. 8, 293–321. 10.1007/BF00992699

[B50] Mnih V. Badia A. P. Mirza M. Graves A. Lillicrap T. Harley T.. (2016). Asynchronousmethods for deep reinforcement learning, in International Conference on Machine Learning (New York, NY), 1928–1937.

[B51] Mnih V. Kavukcuoglu K. Silver D. Graves A. Antonoglou I. Wierstra D.. (2013). Playing atari with deep reinforcement learning. arXiv 1312.5602.25719670

[B52] Mnih V. Kavukcuoglu K. Silver D. Rusu A. A. Veness J. Bellemare M. G.. (2015). Human-level control through deep reinforcement learning. Nature 518, 529–533. 10.1038/nature14236 25719670

[B53] Murray G. M. (2016). Jaw movement and its control, in Functional Occlusion in Restorative Dentistry and Prosthodontics (Elsevier), 55–66. 10.1016/B978-0-7234-3809-0.00005-X

[B54] Muto T. Kanazawa M. (1994). Positional change of the hyoid bone at maximal mouth opening. Oral Surg. Oral Med. Oral Pathol. 77, 451–455. 10.1016/0030-4220(94)90222-4 8028866

[B55] Muto T. Kohara M. Kanazawa M. Kawakami J. (1994). The position of the mandibular condyle at maximal mouth opening in normal subjects. J. Oral Maxillofac. Surg. 52, 1269–1272. 10.1016/0278-2391(94)90049-3 7965330

[B56] Nagabandi A. Kahn G. Fearing R. S. Levine S. (2018). Neural network dynamics for model-based deep reinforcement learning with model-free fine-tuning, in 2018 IEEE International Conference on Robotics and Automation (ICRA) (Long Beach, CA: IEEE), 7559–7566. 10.1109/ICRA.2018.8463189

[B57] Osborn J. (1996). Features of human jaw design which maximize the bite force. J. Biomech. 29, 589–595. 10.1016/0021-9290(95)00117-4 8707785

[B58] Otten E. (2003). Inverse and forward dynamics: models of multi-body systems. Philos. Trans. R. Soc. Lond. B Biol. Sci. 358, 1493–1500. 10.1098/rstb.2003.1354 14561340 PMC1693250

[B59] Ow R. K. K. Carlsson G. E. Karlsson S. (1998). Relationship of masticatory mandibular movements to masticatory performance of dentate adults: a method study. J. Oral Rehabil. 25, 821–829. 10.1046/j.1365-2842.1998.00325.x 9846902

[B60] Peck C. Langenbach G. Hannam A. (2000). Dynamic simulation of muscle and articular properties during human wide jaw opening. Archiv. Oral Biol. 45, 963–982. 10.1016/S0003-9969(00)00071-6 11000383

[B61] Pedersen D. R. Brand R. A. Davy D. T. (1997). Pelvic muscle and acetabular contact forces during gait. J. Biomech. 30, 959–965. 10.1016/S0021-9290(97)00041-9 9302620

[B62] Peng X. B. Abbeel P. Levine S. van de Panne M. (2018). DeepMimic. ACM Trans. Graph. 37, 1–14. 10.1145/3197517.3201311

[B63] Peng X. B. Berseth G. Yin K. Panne M. V. D. (2017). DeepLoco. ACM Trans. Graph. 36, 1–13. 10.1145/3072959.3073602

[B64] Peyron M. A. Lassauzay C. Woda A. (2002). Effects of increased hardness on jaw movement and muscle activity during chewing of visco-elastic model foods. Exp. Brain Res. 142, 41–51. 10.1007/s00221-001-0916-5 11797083

[B65] Posselt U. (1952). Studies in the mobility of the human mandible. Acta Odontol. Scand. 10, 19–160.

[B66] Ruckert E. d'Avella A. (2013). Learned parametrized dynamic movement primitives with shared synergies for controlling robotic and musculoskeletal systems. Front. Comput. Neurosci. 7:138. 10.3389/fncom.2013.00138 24146647 PMC3797962

[B67] Sagl B. Schmid-Schwap M. Piehslinger E. Kronnerwetter C. Kundi M. Trattnig S.. (2019a). *In vivo* prediction of temporomandibular joint disc thickness and position changes for different jaw positions. J. Anat. 234, 718–727. 10.1111/joa.12951 30786005 PMC6481408

[B68] Sagl B. Schmid-Schwap M. Piehslinger E. Kundi M. Stavness I. (2019b). A dynamic jaw model with a finite-element temporomandibular joint. Front. Physiol. 10:1156. 10.3389/fphys.2019.01156 31607939 PMC6757193

[B69] Schulman J. Levine S. Moritz P. Jordan M. Abbeel P. (2015). Trust region policy optimization, in Proceedings of the 32Nd International Conference on International Conference on Machine Learning, Volume 37, ICML'15 (Lille), 1889–1897.

[B70] Schulman J. Wolski F. Dhariwal P. Radford A. Klimov O. (2017). Proximal policy optimization algorithms. arXiv 1707.06347.

[B71] Seireg A. Arvikar R. (1975). The prediction of muscular load sharing and joint forces in the lower extremities during walking. J. Biomech. 8, 89–102. 10.1016/0021-9290(75)90089-5 1150683

[B72] Servin M. Lacoursière C. Melin N. (2006). Interactive simulation of elastic deformable materials, in Proceedings of SIGRAD Conference (Skövde).

[B73] Thelen D. G. Anderson F. C. Delp S. L. (2003). Generating dynamic simulations of movement using computed muscle control. J. Biomech. 36, 321–328. 10.1016/S0021-9290(02)00432-3 12594980

[B74] Tortopidis D. Lyons M. F. Baxendale R. H. Gilmour W. H. (1998). The variability of bite force measurement between sessions, in different positions within the dental arch. J. Oral Rehabil. 25, 681–686. 10.1046/j.1365-2842.1998.00293.x 9758398

[B75] Tsuruta J. Mayanagi A. Miura H. Hasegawa S. (2002). An index for analysing the stability of lateral excursions. J. Oral Rehabil. 29, 274–281. 10.1046/j.1365-2842.2002.00904.x 11896845

[B76] Van Hasselt H. Guez A. Silver D. (2016). Deep reinforcement learning with double q-learning, in Thirtieth AAAI Conference on Artificial Intelligence (Phoenix, AZ).

[B77] Vigotsky A. D. Halperin I. Lehman G. J. Trajano G. S. Vieira T. M. (2018). Interpreting signal amplitudes in surface electromyography studies in sport and rehabilitation sciences. Front. Physiol. 8:985. 10.3389/fphys.2017.00985 29354060 PMC5758546

[B78] Xu W. Bronlund J. Potgieter J. Foster K. Röhrle O. Pullan A. (2008). Review of the human masticatory system and masticatory robotics. Mech. Mach. Theory 43, 1353–1375. 10.1016/j.mechmachtheory.2008.06.003

[B79] Yin H. H. Knowlton B. J. (2006). The role of the basal ganglia in habit formation. Nat. Rev. Neurosci. 7:464–476. 10.1038/nrn1919 16715055

